# A first-in-human study of JNJ-70218902, a bispecific T-cell-redirecting antibody against TMEFF2 in metastatic castration-resistant prostate cancer

**DOI:** 10.1093/oncolo/oyae313

**Published:** 2025-01-20

**Authors:** Emiliano Calvo, Bernard Doger, Joan Carles, Avivit Peer, David Sarid, Bernhard J Eigl, Anjali Avadhani, David Yao, Vincent Lin, Shujian Wu, Pharavee Jaiprasart, John Loffredo, Monelle Tamegnon, Weichun Xu, Hong Xie, Aaron R Hansen

**Affiliations:** START Madrid-CIOCC, Centro Integral Oncológico Clara Campal, 28050 Madrid, Spain; START Madrid-FJD, Hospital Universitario Fundación Jiménez Díaz, 28040 Madrid, Spain; Vall d’Hebron Institute of Oncology, Vall d’Hebron University Hospital, 08035 Barcelona, Spain; Rambam Health Care Campus, Haifa, 3109601, Israel; Tel Aviv Sourasky Medical Center, Tel Aviv, 6423906, Israel; BC Cancer – Vancouver, Vancouver, BC V5Z 4E6, Canada; Janssen R&D, Raritan, NJ, 08869, United States; Janssen R&D, Raritan, NJ, 08869, United States; Janssen R&D, Raritan, NJ, 08869, United States; Janssen R&D, Raritan, NJ, 08869, United States; Janssen R&D, Raritan, NJ, 08869, United States; Janssen R&D, Raritan, NJ, 08869, United States; Janssen R&D, Raritan, NJ, 08869, United States; Janssen R&D, Raritan, NJ, 08869, United States; Janssen R&D, Raritan, NJ, 08869, United States; Department of Medical Oncology, Princess Margaret Hospital, Toronto, ON M5G 2M9, Canada; Department of Medicine, University of Queensland, Brisbane, QLD 4072, Australia

**Keywords:** castration-resistant prostate cancer, immunotherapy, phase I, prostate cancer, T-lymphocytes

## Abstract

**Background:**

Metastatic castration-resistant prostate cancer (mCRPC) has a poor prognosis, necessitating the investigation of novel treatments and targets. This study evaluated JNJ-70218902 (JNJ-902), a T-cell redirector targeting transmembrane protein with epidermal growth factor-like and 2 follistatin-like domains 2 (TMEFF2) and cluster of differentiation 3, in mCRPC.

**Patients and methods:**

Patients who had measurable/evaluable mCRPC after at least one novel androgen receptor–targeted therapy or chemotherapy were eligible. Participants received subcutaneous JNJ-902 0.3, 1.0, 1.5, 3.0, or 6.0 mg once weekly (QW) or 2.0, 3.0, 4.0, or 6.0 mg biweekly (Q2W). Study objectives included assessment of safety, pharmacokinetics, immunogenicity, and preliminary efficacy.

**Results:**

Eighty-two participants were enrolled to receive at least one dose of JNJ-902 (QW; *n *= 38; Q2W; *n *= 44). Median duration of treatment was 1.91 (0.0-19.4) months across dosing groups. All participants experienced at least one treatment-emergent adverse event (TEAE) and 76 (92.7%) experienced treatment-related TEAEs. Fourteen participants (17.1%) experienced a TEAE that led to study discontinuation, of which 3 (3.7%) were related to JNJ-902. Dose-limiting toxicities were observed in 2 participants (2.4%). Five participants (15.2%) with measurable disease had a confirmed partial response and 10 participants (12.2%) had ≥50% decrease from baseline prostate-specific antigen levels. Clinical activity was not dose related and no clear exposure-response relationship was observed.

**Conclusions:**

In this study, dose escalation was limited by emerging dose-limiting toxicities. Although a recommended phase II dose was not determined, findings indicate TMEFF2 to be a potential target in mCRPC that warrants further investigation.

**Implications for practice**

Despite advances in treatment, metastatic castration-resistant prostate cancer continues to carry a poor prognosis and survival rate, resulting in an unmet need for novel targets and treatment modalities. Preclinical efficacy of JNJ-70218902, a bispecific T-cell redirecting antibody against TMEFF2, and a TMEFF2-targeting antibody-drug conjugate indicate TMEFF2 as a potential target for prostate cancer. This first-in-human study with JNJ-70218902 showed clinical activity, although dose escalation was limited by dose limiting toxicities and showed no clear exposure-response relationship. Notwithstanding limitations, the data support further exploration of TMEFF2 as a therapeutic target for metastatic castration-resistant prostate cancer.

## Introduction

Prostate cancer carries a global health burden with more than 1.4 million new cases reported worldwide in 2020.^1^ Although only ~8% of newly diagnosed prostate cancer cases in the US are metastatic prostate cancer, one-third of early-stage prostate cancer tumors recur with treatment and 10%-20% of patients develop metastatic castration-resistant prostate cancer (mCRPC) within 5 years of follow-up.^2-4^ Current treatment options for mCRPC include chemotherapy and androgen receptor (AR) pathway inhibitors and, more recently, targeted radioligand therapy and PARP inhibitor monotherapy or combinations for a subset of patients with altered DNA repair genes.^5,6^ Despite advances in treatment, mCRPC continues to carry a poor prognosis, with a median survival less than 3 years in phase 3 studies and less than 2 years in the real-world setting.^5,7^ As such, there remains an unmet medical need to investigate both new treatment targets and novel treatment modalities that do not overlap with existing treatment options.

T-cell redirection using bispecific antibodies has been suggested to be a promising novel treatment approach for mCRPC, as these agents can enhance immunoreactivity in an otherwise immunologically “cold” tumor microenvironment (TME).^8^ Bispecific antibodies have already been approved for the treatment of hematologic malignancies and are being investigated in prostate cancer with agents predominantly targeting prostate-specific membrane antigen (PSMA).^9-14^ However, PSMA is the target of several novel therapies, such as radiopharmaceuticals or antibody-drug conjugates, that may impact treatment sequencing with these agents. In addition, PSMA expression is regulated by AR signaling, which is modulated by several prostate cancer therapies as part of disease management.^15,16^ Accordingly, additional targets are needed to support the introduction of novel therapies that are less dependent on AR signaling, DNA repair, or PSMA, and expand the mCRPC treatment landscape.

The transmembrane protein with epidermal growth factor-like and 2 follistatin-like domains 2 (TMEFF2) is primarily expressed in prostate tissue and the brain.^17-19^ TMEFF2 functions are not fully understood, with some reports indicating a role in embryonic development; while TMEFF2 knockout mice appeared to be normal at birth, these mice stopped thriving at 3 weeks of age.^20,21^ TMEFF2 has been reported to have both oncogenic and tumor-suppressive roles depending on tumor type or prostate cancer model system.^17,20,22,23^ TMEFF2 regulation also remains unclear, where some studies indicate TMEFF2 expression to be androgen independent while others suggest that it is androgen dependent.^20^

Notably, TMEFF2 is found to be highly expressed and prevalent in tumors across prostate cancer subtypes, including those transitioning to neuroendocrine features, thereby enabling this antigen to serve as an “address” for T-cell redirector treatment.^18,19,24^ In later stage disease settings, TMEFF2 expression was observed in 41 of 45 (91%) metastatic castration-resistant prostate cancer (mCRPC) tissue samples.^24^ JNJ-70218902 (JNJ-902) is a bispecific antibody built with the DuoBody technology that selectively binds to both TMEFF2 on tumor cells and the cluster of differentiation 3 (CD3) complex on T cells. This dual binding redirects T cells to TMEFF2-expressing tumor cells, leading to T-cell activation and target tumor cell lysis.^24^ Prostate cancer mouse models and patient-derived xenografts treated with JNJ-902 showed a decrease in mean tumor volume that was associated with intratumoral infiltration of T cells.^24^ Taken together, these data support further investigation of JNJ-902 for the treatment of prostate cancer.

The aim of this first-in-human study was to evaluate the safety, pharmacokinetics (PK), and preliminary clinical activity of JNJ-902 in participants with previously treated mCRPC.

## Materials and methods

### Study design and participants

This first-in-human, open-label, multicenter, phase I study evaluated JNJ-902 monotherapy in participants with mCRPC in Canada, Israel, and Spain. The study was designed to be conducted in 2 parts: dose escalation (part 1) and dose expansion (part 2). Dose expansion was not initiated and is not described in this article. Planned biomarker parameters were not analyzed at the time of this manuscript submission.

Participants ≥18 years of age with measurable or evaluable mCRPC and histologically confirmed adenocarcinoma were eligible for enrollment. Demonstration of TMEFF2 protein expression in prostate cancer tissue was not required for eligibility. Eligible participants had an Eastern Cooperative Oncology Group (ECOG) performance status ≤1 and had received prior treatment with at least one novel AR-targeted therapy or chemotherapy. Participants who had not undergone prior orchiectomy, and thus were receiving gonadotropin-releasing hormone (GnRH) agonists or antagonist analogs, must have initiated GnRH treatment prior to the first study dose and continued treatment throughout the study period. Participants with a known history of brain metastases were excluded, and the protocol included management guidelines and monitoring of neurological toxicities due to concerns of TMEFF2 expression in normal brain tissue. Participants with clinically significant pulmonary compromise, peripheral neuropathy, neuropathic pain of grade ≥2, or cardiovascular abnormalities within 6 months prior to informed consent were also excluded. Concurrent use of other anticancer treatments or an investigational agent for the treatment of advanced prostate cancer was prohibited.

Participants received a dose of JNJ-902 at 0.3 mg, 1.0 mg, 1.5 mg, 3.0 mg, or 6.0 mg once-weekly (QW), or at 2.0 mg, 3.0 mg, 4.0 mg, or 6.0 mg once every 2 weeks (Q2W), administered via subcutaneous (SC) injection. Intrapatient dose escalation was permitted. Participants were hospitalized for a minimum of 36 hours following the first treatment dose and any step-up dose(s) of JNJ-902. Treatment with a corticosteroid, antihistamine, and antipyretic was required prior to the first 2 SC doses of JNJ-902 and subsequently repeated if there were cytokine release syndrome (CRS) or systemic administration-related reaction events with the previous dose. Participants continued to receive treatment until radiographic or clinical disease progression, unacceptable toxicity, withdrawal of consent, or loss to follow-up.

This study (NCT04397276) was conducted with the approval of each study site’s Independent Ethics Committee/Institutional Review Board, and in accordance with the Declaration of Helsinki, the US Food & Drug Administration Investigational New Drug regulations, Good Clinical Practice guidelines, and any applicable regulatory requirements. Patients or their legal representatives provided written informed consent to participate in the study.

### Outcomes and assessments

The primary endpoints of part 1 of the study were incidence and severity of all adverse events (AEs), including incidence of dose-limiting toxicity (DLT), to determine the recommended phase 2 dose (RP2D) and maximum tolerated dose. Safety was evaluated through AEs, clinical laboratory tests, electrocardiograms, physical and neurological examinations, ophthalmological examinations, and assessments of vital signs and ECOG performance status. Neurological examinations were performed by the investigator at baseline and on every treatment day throughout the study for early recognition of neurological AEs. AEs, except for CRS, were graded using Common Terminology Criteria for Adverse Events version 5.0 (CTCAE v5.0). CRS events were graded using the American Society for Transplantation and Cellular Therapy guidelines.^25^ DLTs were assessed during the first 21 days of treatment. DLT criteria included grade ≥4 neutropenia or thrombocytopenia for at least 7 days, or grade ≥3 non-hematological toxicity. Full DLT criteria are outlined in Supplementary Table S1.

Secondary objectives included assessment of PK parameters; immunogenicity, assessed by presence of anti-JNJ-902 antibodies; and preliminary clinical activity, assessed as objective response rate and duration of response according to response criteria of Prostate Cancer Working Group 3 or Response Evaluation Criteria in Solid Tumors version 1.1 by the investigator, prostate-specific antigen (PSA) response rate, and time to response. Radiographic response was assessed using computed tomography (CT) scans or magnetic resonance imaging (MRI), and whole-body bone scans. CT, MRI, and whole-body bone scans were performed every 8 (±1) weeks after the first dose for the first 24 weeks, followed by every 12 (±1) weeks thereafter. PSA levels were assessed every 4 (±1) weeks after the first dose.

Blood samples were collected to assess serum concentrations of JNJ-902 and anti-JNJ-902 antibodies to analyze PK and immunogenicity, respectively. Safety, PK, and immunogenicity data were monitored at each dose escalation and at regular intervals throughout the study period.

### Statistical analysis

Sequential dose escalations were guided by the modified continual reassessment method based on the Bayesian logistic regression model with the escalation with overdose control principle. The total number of participants enrolled was dependent on the frequency of DLT and identification of the RP2D. Data were summarized using descriptive statistics.

## Results

### Study population

Eighty-two males with mCRPC were enrolled and treated with at least one dose of JNJ-902 (overall population), with 38 (46.3%) and 44 (53.7%) participants receiving QW or Q2W doses, respectively. Most participants were White (78 [95.1%]), with a median age of 66.0 years (range, 50-84) and a median baseline PSA level of 68.19 µg/L (range, 0.0-2115.0). All participants had previously been treated with at least one systemic therapy. Most participants received prior treatment with a novel AR-targeted therapy (81 [98.8%]) and chemotherapy (79 [96.3%]), including docetaxel (78 [95.1%]) or cabazitaxel (50 [61.0%]). Participant demographics and baseline characteristics were broadly comparable across dosing groups (not shown) and are summarized for the overall population in Table 1.

**Table 1.** Participant demographics and baseline characteristics.

Participant demographic	Overall (*N* = 82)
Age, median (range), years	66.0 (50-84)
≤65, *n* (%)	37 (45.1)
≥65, *n* (%)	45 (54.9)
Sex, *n* (%)	
Male	82 (100)
Race, *n* (%)	
Asian	2 (2.4)
White	78 (95.1)
Multiple	1 (1.2)
Unknown	1 (1.2)
Ethnicity, *n* (%)	
Hispanic or Latino	2 (2.4)
Not Hispanic or Latino	50 (61.0)
Unknown	30 (36.6)
ECOG, *n* (%)	
0	34 (41.5)
1	48 (58.5)
Extent of disease, *n* (%)	
Bone	76 (92.7)
Soft tissue	59 (72.0)
Visceral^a^	35 (42.7)
Lymph node^b^	42 (51.2)
Other	10 (12.2)
PSA at baseline, µg/L	
Mean (SD)	242.27 (399.176)
Median (range)	68.19 (0.0–2115.0)
Prior cancer-related therapy, n (%)	
Prostatectomy	28 (34.1)
Radiotherapy	57 (69.5)
Hormonal therapy	82 (100)
Orchiectomy	1 (1.2)
GnRHa	75 (91.5)
First-generation antiandrogen	31 (37.8)
AR pathway inhibitors	81 (98.8)
Other hormonal therapy	1 (1.2)
Chemotherapy	79 (96.3)
Docetaxel	78 (95.1)
Cabazitaxel	50 (61.0)
Other chemotherapy	19 (23.2)
Other	34 (41.5)

^a^Included lungs, liver, adrenal glands, and central nervous system.

^b^Included pelvic and extra-pelvic.

Abbreviations: AR, androgen receptor; ECOG, Eastern Cooperative Oncology Group; GnRHa, gonadotropin-releasing hormone analog; PSA, prostate-specific antigen.

The median duration of JNJ-902 treatment across all cohorts was 1.91 months (range, 0.0-19.4). Dose increase occurred in 5 participants (6.1%), dose reduction in 7 participants (8.5%), commonly due to AEs, and delayed injections in 39 participants (47.6%). At the time of the database lock (January 18, 2023), 5 participants (6.1%) were still receiving treatment. Reasons for discontinuing treatment included disease progression (60 [73.2%]), AEs (6 [7.3%]), death due to disease progression (5 [6.1%]), participant refusal of further study treatment (4 [4.9%]), and physician decision (2 [2.4%]).

### Safety

All 82 participants experienced at least one treatment-emergent adverse event (TEAE) of any grade, which included fatigue (44 [53.7%]), injection site erythema (41 [50.0%]), decreased appetite (38 [46.3%]), anemia (30 [36.6%]), back pain (23 [28.0%]), and weight decreased (20 [24.4%]) (Supplementary Table S2). Forty-six participants (56.1%) experienced a grade ≥3 TEAE, including anemia (14 [17.1%]) and fatigue (10 [12.2%]) (Supplementary Table S2). TEAEs occurring in ≥10% of the overall population by preferred term are summarized in Supplementary Table S2. Overall, the incidence of TEAEs was similar across all dosing cohorts, with 20 participants (52.6 %) in the QW cohorts and 26 (59.1%) in the Q2W cohorts experiencing grade ≥3 TEAEs (not shown).

Four participants (4.9%) (1 participant [1.2%] each in the 1.5 mg QW and 6.0 mg QW dosing cohorts, and 2 participants [2.4%] in the 6.0 mg Q2W dosing cohort) experienced at least one treatment-emergent CRS event. The most common CRS events included pyrexia in all 4 participants, and chills and hypotension in 2 participants each (2.4%). All CRS events were low grade; however, 2 CRS events were considered serious. Participants were treated with analgesics and antipyretics for management of CRS.

Treatment-related TEAEs were reported in 76 (92.7%) participants (Table 2), which included injection site erythema (41 [50.0%]), decreased appetite (31 [37.8%]), fatigue (29 [35.4%]), injection site pruritus (18 [22.0%]), and weight decreased (18 [22.0%]) (Table 3). Seven (8.5%) and 15 (18.3%) participants experienced a serious and grade ≥3 treatment-related TEAE, respectively (Table 2). The most common grade ≥3 treatment-related TEAEs were fatigue (5 [6.1%]), lymphopenia (5 [6.1%]), asthenia (3 [3.7%]), and arthralgia (2 [2.4%]) (Table 3). Treatment-related TEAEs occurring in ≥5% of the overall population by preferred term are summarized in Table 3.

**Table 2.** Overview of TEAEs in the all-treated analysis set.

Participants with events, *n* (%)	QW SC dosing (mg)						Q2W SC dosing (mg)					
	0.3	1	1.5	3	6	Total	2	3	4	6	Total	Overall
Participants, *n*	2	6	12	11	7	38	5	21	12	6	44	82
≥1 TEAE	2 (100)	6 (100)	12 (100)	11 (100)	7 (100)	38 (100)	5 (100)	21 (100)	12 (100)	6 (100)	44 (100)	82 (100)
Treatment-related TEAEs^a^	2 (100)	6 (100)	12 (100)	10 (90.9)	6 (85.7)	36 (94.7)	5 (100)	19 (90.5)	10 (83.3)	6 (100)	40 (90.9)	76 (92.7)
Grade ≥3 TEAEs	2 (100)	0	6 (50.0)	6 (54.5)	6 (85.7)	20 (52.6)	3 (60.0)	9 (42.9)	10 (83.3)	4 (66.7)	26 (59.1)	46 (56.1)
Treatment-related grade ≥3 TEAEs^a^	0	0	2 (16.7)	2 (18.2)	2 (28.6)	6 (15.8)	1 (20.0)	1 (4.8)	6 (50.0)	1 (16.7)	9 (20.5)	15 (18.3)
TEAEs leading to death^b^	0	0	0	1 (9.1)	0	1 (2.6)	0	1 (4.8)	0	0	1 (2.3)	2 (2.4)
Treatment-related TEAEs leading to death^a^^,^^b^	0	0	0	0	0	0	0	0	0	0	0	0
Serious TEAEs	2 (100)	1 (16.7)	3 (25.0)	5 (45.5)	6 (85.7)	17 (44.7)	3 (60.0)	8 (38.1)	5 (41.7)	3 (50.0)	19 (43.2)	36 (43.9)
Treatment-related serious TEAEs^a^	0	1 (16.7)	1 (8.3)	1 (9.1)	2 (28.6)	5 (13.2)	0	0	1 (8.3)	1 (16.7)	2 (4.5)	7 (8.5)
TEAEs leading to discontinuation of study agent	1 (50.0)	1 (16.7)	1 (8.3)	1 (9.1)	1 (14.3)	5 (13.2)	1 (20.0)	5 (23.8)	2 (16.7)	1 (16.7)	9 (20.5)	14 (17.1)
Treatment-related TEAEs leading to discontinuation of study agent^a^	0	0	0	0	1 (14.3)	1 (2.6)	0	0	1 (8.3)	1 (16.7)	2 (4.5)	3 (3.7)

^a^Assessed by the investigator as related to study agent.

^b^AEs leading to death are based on AE outcome of Fatal.

A TEAE was defined as any AE with onset date and time on or after that of the first dose through 30 days after the last dose of study drug or the day prior to start of subsequent therapy, whichever is earlier. Any AE that was assessed by the investigator as related to study drug was also considered treatment-emergent regardless of onset day.

Abbreviations: AE, adverse event; QW, once weekly; Q2W, once every 2 weeks; SC, subcutaneous; TEAE, treatment-emergent adverse event.

**Table 3.** Treatment-related TEAEs in ≥5% of participants by preferred term in the overall population.

Participants, *n* (%)	All grades	≥ Grade 3
Overall treatment-related TEAEs	76 (92.7)	15 (18.3)
Treatment-related TEAEs in ≥5% of participants		
Injection site erythema	41 (50.0)	0
Decreased appetite	31 (37.8)	1 (1.2)
Fatigue	29 (35.4)	5 (6.1)
Injection site pruritus	18 (22.0)	0
Weight decreased	18 (22.0)	0
Injection site rash	14 (17.1)	0
Dysgeusia	12 (14.6)	0
Nausea	10 (12.2)	0
Arthralgia	9 (11.0)	2 (2.4)
Pyrexia	7 (8.5)	0
AST increased	7 (8.5)	1 (1.2)
Pruritus	7 (8.5)	0
Asthenia	6 (7.3)	3 (3.7)
Headache	6 (7.3)	0
Vomiting	6 (7.3)	0
Lymphopenia	6 (7.3)	5 (6.1)
Hypotension	6 (7.3)	1 (1.2)
Injection site reaction	5 (6.1)	0
ALT increased	5 (6.1)	1 (1.2)
Hypesthesia	5 (6.1)	0
Myalgia	5 (6.1)	0

Treatment-related TEAEs were assessed by the investigator and deemed related to study drug.

Participants were counted only once for any given event, regardless of the number of times they experienced the event.

Abbreviations: ALT, alanine aminotransferase; AST, aspartate aminotransferase; TEAE, treatment-emergent adverse event.

Thirty-six participants (43.9%) experienced at least 1 serious TEAE across all cohorts (Table 2). Seven participants (8.5%) had a serious TEAE that was related to JNJ-902 (Table 2), including vomiting and confusional state (2 participants each [2.4%]), alanine aminotransferase (ALT) increased, aspartate aminotransferase (AST) increased, asthenia, balance disorder, CRS, fall, hyperbilirubinemia, and orthostatic hypotension (one participant each [1.2%]).

Forty-six (56.1%) participants experienced at least 1 TEAE of any grade grouped under the CTCAE v 5.0 System Organ Class (SOC) nervous system disorders, with dysgeusia experienced by ≥10% of participants (12 [14.6%]) (Supplementary Table S2). Within the nervous system disorders SOC, 9 (11.0%) participants experienced a grade ≥3 TEAE (*n* = 2 each: dizziness, spinal cord compression, and syncope; *n* = 1 each: cerebrovascular accident, medullary compression syndrome, and presyncope) and 7 (8.5%) participants experienced a serious TEAE (*n* = 2 each: spinal cord compression; *n* = 1 each: balance disorder, cerebrovascular accident, cervical radiculopathy, hemianopia, and medullary compression syndrome). Treatment-related TEAEs grouped under the nervous system disorders SOC were reported in 28 participants (34.1%), of which 2 (2.4%) experienced a grade 3 event of dizziness (1 [1.2%]) and syncope (1 [1.2%]).

Fourteen (17.1%) participants experienced a TEAE that led to study discontinuation, including 8 (9.8%) with grade ≥3 TEAEs (data not shown) and 3 (3.7%) with TEAEs considered treatment-related (Table 2). Treatment-related TEAEs leading to discontinuation included orthostatic hypotension (1 participant each in the 6 mg QW and 6 mg Q2W dosing cohorts) and fatigue (1 participant in the 4 mg Q2W dosing cohort). Fifty (61.0%) participants reported TEAEs that led to dose interruption or reduction. Seven (8.5%) deaths were reported during the study, 5 (6.1%) due to disease progression, and 2 (2.4%) due to AEs that were deemed unrelated to JNJ-902.

Clinical laboratory tests revealed a shift of at least one grade from baseline in lymphocyte count decrease in 51 participants (62.1%). For other hematology parameters, the most notable was a shift in anemia of 2 grades in 9 (11.0%) participants. For chemistry laboratory parameters, AST increased was the most frequent shift, with an increase of at least one grade from baseline in 20 participants (24.4%).

Two participants experienced TEAEs at the 6 mg dose level that were considered DLTs, all of which were grade 3. An 81-year-old participant in the 6 mg QW cohort experienced a DLT of fall after 3 doses, which led to dose reduction. This participant subsequently developed orthostatic hypotension while on a reduced dose and was eventually withdrawn from the study after receiving two additional doses. The other 81-year-old participant in the 6 mg Q2W cohort experienced orthostatic hypotension followed by syncope after the first dose. Syncope resolved after withdrawal of JNJ-902, but orthostatic hypotension was not resolved as of study cutoff. The observed DLTs at 6 mg precluded further dose escalations and consequently an RP2D could not be determined.

### Pharmacokinetics

**Figure 1.**
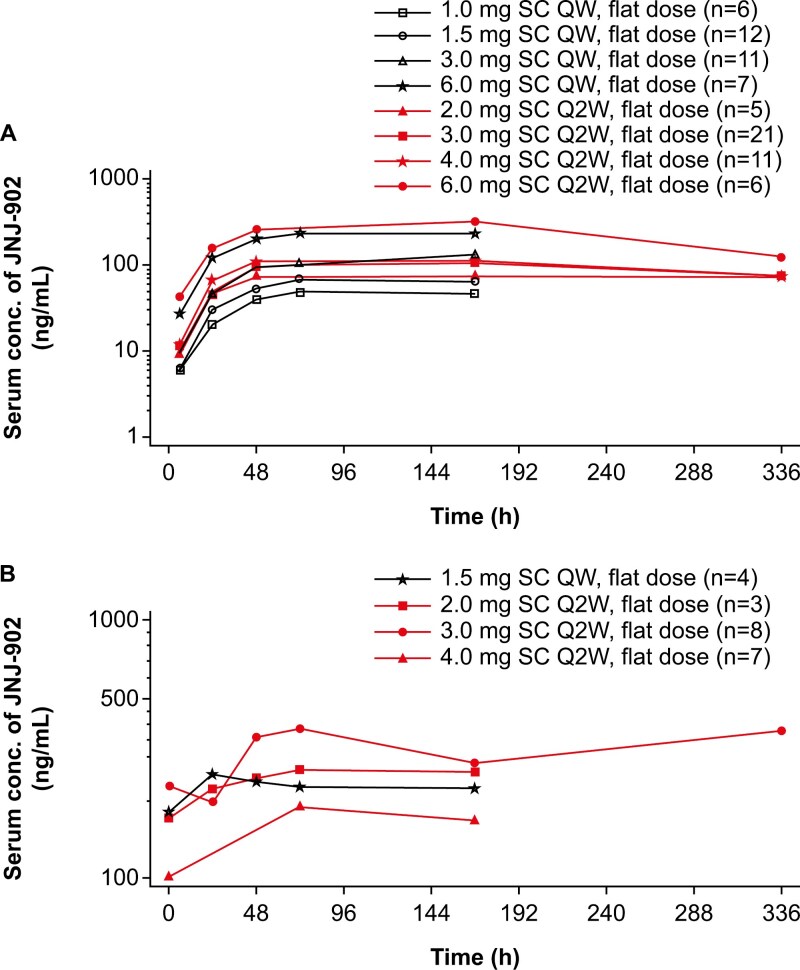
Mean serum concentration-time profiles of JNJ-902. (A) Mean serum concentration following the first dose of the repeated SC administration. JNJ-902 was administered at 0.3 mg, 1.0 mg, 1.5 mg, 3.0 mg, and 6.0 mg QW (black lines) and 2.0 mg, 3.0 mg, 4.0 mg, and 6.0 mg Q2W (red lines). 0.3 mg QW cohort with *n* < 3 is not shown. (B) Mean serum concentration following repeated SC administrations. Serum concentrations of the fourth Q2W dose were assessed for 2.0 mg, 3.0 mg, and 4.0 mg (red lines) SC administrations. Serum concentrations during the seventh QW dose were available for 1.5 mg SC administrations (black line). 3.0 mg QW at dose 7 with *n* < 3 is not shown. (A-B) Y-axes are presented in semi-logarithmic scale. Abbreviations: conc, concentration; h, hours; *n*, number of participants with pharmacokinetic data in each cohort.

PK parameters were analyzed in all 82 participants. After the first dose of the repeated SC QW administration, JNJ-902 exposures in serum (maximum JNJ-902 serum concentration [*C*_max_] and area under the JNJ-902 serum concentration-time curve [AUC] from time 0 to 168 hours [AUC_168h_]) had an approximately dose-proportional increase for all doses, with the mean *C*_max_ ranging from 51.7 ng/mL (1.0 mg cohort) to 249 ng/mL (6.0 mg cohort) (Figure 1A, Supplementary Table S3). JNJ-902 serum concentration-time profiles showed a similar pattern after the first Q2W dose, with approximately dose-proportional *C*_max_ and AUC_336h_ with increasing JNJ-902 doses (Figure 1A, Supplementary Table S3). The median time to reach peak maximum concentration (*t*_max_) was approximately 3-7 days across all dose levels (Supplementary Table S3).

Steady state was achieved after the seventh QW dose of 1.5 mg and the fourth Q2W dose of 2.0 mg, 3.0 mg, or 4.0 mg JNJ-902 with repeated administration (Figure 1B). Median *t*_max_ at doses 7 and 4 was approximately 2-3 days, with a mean ratio of accumulation ranging from 2.17 to 4.45 across dose levels (Supplementary Table S4).

### Immunogenicity

Three of 79 (3.8%) participants from the 1.5 mg and 3.0 mg QW cohorts and the 4.0 mg Q2W cohort were positive for anti-drug antibodies, with peak titers ranging from 1:10 to 1:20. ADAs did not appear to have any impact on safety or PK.

### Preliminary efficacy

**Figure 2.**
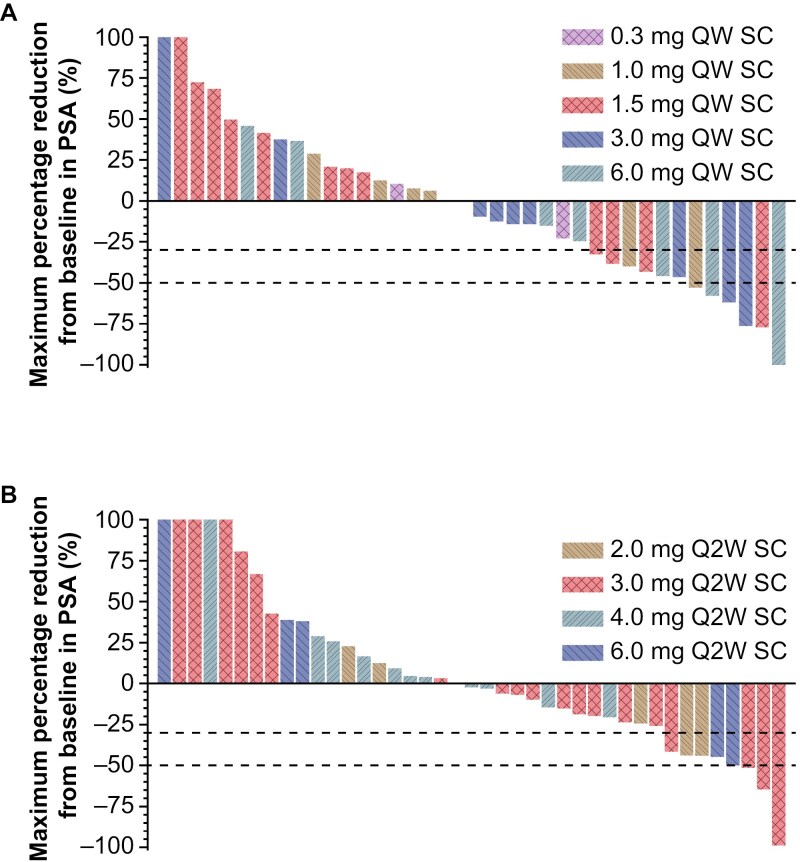
Maximum percentage reduction from baseline in PSA: all-treated analysis set. (A) Percentage PSA change from baseline in participants receiving 0.3 mg, 1.0 mg, 1.5 mg, 3.0 mg, and 6.0 mg QW SC administration of JNJ-902 (*n* = 38). (B) Percentage PSA change from baseline in participants receiving 2.0 mg, 3.0 mg, 4.0 mg, and 6.0 mg Q2W SC administration of JNJ-902 (*n* = 41). (A-B) Dashed lines represent 30% and 50% decreases. Increases greater than 100% are set to 100%. *n* = number of participants with both baseline and at least one post-baseline assessment.

Five of 33 (15.2%) participants with measurable disease per Response Evaluation Criteria in Solid Tumors version 1.1 had a confirmed partial response with a duration of response ranging from 4.5 to 15.4 months. Time to first response was less than 2 months for 3 participants, with 2 participants showing response lasting over 9 months. Additionally, 10 of the 82 (12.2%) participants treated with JNJ-902 (6 in the QW cohorts and 4 in the Q2W cohorts) had ≥50% decrease from baseline PSA levels (Figure 2). However, a clear exposure-response relationship was not observed.

## Discussion

This first-in-human study was initiated to investigate the safety, PK, and preliminary activity of JNJ-902, a novel T-cell redirector targeting the potential prostate cancer target TMEFF2 in 82 patients with mCRPC. As the dose of JNJ-902 was escalated to 6 mg, 2 participants experienced DLTs of a suspected neurologic nature, one of which occurred in the setting of a CRS event. Accordingly, given the neurologic nature of these DLTs, an effect of immune effector cell-associated neurotoxicity syndrome (ICANS) could not be ruled out. The observed DLTs at 6 mg did not meet the prespecified DLT target rate but precluded further dose escalations, as participants with mCRPC were generally older and commonly hypertensive, and thus were taking antihypertensive medications, thereby increasing the risk for AEs related to orthostatic hypotension. Consequently, the AE profile of JNJ-902, such as incidence or severity of CRS, ICANS, and neurological effects, could not be fully characterized, and an RP2D could not be determined. The limited dose levels assessed in this study may have contributed to the lack of a clear exposure-response relationship. However, despite the limited dose escalation, 5 participants (15.2%) with measurable disease experienced a confirmed partial response. Additionally, time to first response was less than 2 months for 3 participants, with 2 participants showing response lasting over 9 months. Nevertheless, there was no clear exposure-response relationship to PSA and further investigation of JNJ-902 will not be pursued in this treatment setting.

Due to TMEFF2 expression in normal brain tissue, strategies to promote early recognition of neurological AEs included treatment surveillance with neurological examinations at baseline and on every treatment day throughout the study. More than half of the participants in various dose cohorts were found to have experienced at least one TEAE grouped under nervous system disorders. Two participants (2.4%) experienced treatment-related grade 3 TEAEs grouped under nervous system disorders, including dizziness in one and syncope in the other. Both the dizziness and syncope events were deemed not associated with CRS or ICANs. TEAEs of dizziness, syncope, and orthostatic hypotension could suggest a cardiovascular etiology. Cardiovascular events including hypotension, left ventricular dysfunction, and arrythmias have been reported with CAR-T therapy and T-cell engagers, often, but not always, in association with CRS.^26,27^ However cardiac testing was unrevealing in the cases reported for JNJ-902, and the timing of the events and occurrence of other neurologic treatment related TEAEs in the study, including paresthesia, dysesthesia, balance disorder, and abnormal coordination suggests a potential neurological etiology. Given the small number of cases and diverse events reported, it is difficult to ascribe a mechanism or associate these TEAE with TMEFF2 expression in the nervous system. Further study of TMEFF2 function in the nervous system may illuminate the clinical findings in this study.

Due to the mechanism of action of JNJ-902 in binding and activating T cells, and the subsequent release of cytokines, CRS events were anticipated. In addition, other bispecific agents currently being investigated in mCRPC have demonstrated all-grade and grade 3 CRS events in 63%-84% and 4%-31% of participants, respectively.^13,21^ Although CRS was anticipated as a risk for JNJ-902, CRS events were not frequently observed in this study potentially due to the limited dose escalation, although other factors, such as subcutaneous administration, may have contributed. CRS events were observed at 1.5 mg QW, 6.0 mg QW, and 6.0 mg Q2W dosing regimen and were of low grade (all grade ≤2).

While time to first response was relatively rapid (<2 months for 3 participants), response rates were modest (15.2%). Other bispecific CD3-redirecting agents have also shown modest response rates (5.6%-14.3% partial response) in mCRPC,^13,28^ which may reflect tumor biology or the TME. Challenges associated with CD3+ T-cell redirection in cancer have been suggested to include the recruitment of counterproductive CD3+ T-cell subsets, the presence of an immunosuppressive TME, T-cell dysfunction and exhaustion due to expression of immune checkpoint molecules, and tumor antigen escape.^29^ Accordingly, further research to better understand if and how bispecific agents can circumvent these challenges in mCRPC is necessary.

Notably, this study has a few inherent limitations due to the nature of phase I first-in-human studies in mCRPC. This includes enrolling participants who have been heavily pretreated and have received prior treatment with chemotherapy and coadministration of corticosteroids, which can impact the immune system, thereby influencing the response to immunotherapies. Additionally, mCRPC is heterogeneous in nature, and previous studies have suggested immunotherapy to be effective in certain subsets of participants with mCRPC.^30-33^ As such, identification of biomarkers that correlate with response to T-cell redirectors may help guide patient selection.

Tumors from patients enrolled in this trial were almost all positive for TMEFF2 expression using an IHC assay on archival tissue samples (data not shown), in agreement with preclinical data.^24^ However, due to the limited dose escalations, small cohort sizes, and the lack of a clear exposure-response relationship, no association could be made between TMEFF2 expression and safety or efficacy of JNJ-902. Other biomarker assessments were similarly inconclusive or not performed.

The clinical activity observed in this first-in-human study indicates that TMEFF2 may be a potential therapeutic target for prostate cancer. To confirm the candidacy of TMEFF2 as a prostate cancer target, the biological and pathological functions of TMEFF2 need to be further investigated.

## Summary

In conclusion, due to the limited dose escalation in this study, an RP2D could not be determined. However, clinical activity was observed despite limited dosing levels. Taken together, data from this study demonstrate that therapeutic approaches targeting TMEFF2 should be further explored.

Further efforts are necessary to better characterize the role and regulation of TMEFF2 in normal tissues and prostate cancer and identify the best model systems to allow translation of preclinical efficacy to patient benefit.

## Supplementary Material

oyae313_suppl_Supplementary_Tables_1-4

## Data Availability

The data sharing policy of Janssen Pharmaceutical Companies of Johnson & Johnson is available at https://yoda.yale.edu/about/data-holders/johnson-johnson/. Requests for access to the study data can be submitted through the Yale Open Data Access (YODA) Project site at http://yoda.yale.edu
