# Supplementary material for: A first-in-human study of JNJ-70218902, a bispecific T-cell-redirecting antibody against TMEFF2 in metastatic castration-resistant prostate cancer
Source: Oncologist. 2025 Jan 20;30(1):oyae313. doi: 10.1093/oncolo/oyae313 (PMC11745015; doi:10.1093/oncolo/oyae313)
Supplement: oyae313_suppl_Supplementary_Tables_1-4 [file oyae313_suppl_supplementary_tables_1-4.docx]

**Supplementary Data**

**Tables**

**Supplementary Table S1.** Dose-limiting toxicity criteria^a^

| **Hematologic toxicity** | |
| --- | --- |
| Neutrophil count decreased | Febrile neutropenia |
|  | Neutropenia: grade 4 for ≥7 days |
| Platelet count decreased | Grade ≥3 thrombocytopenia with bleeding or any grade 4 thrombocytopenia with duration ≥7 days |
| Any hematological toxicity | Grade 5 |
| **Non-hematological toxicity** | |
| Any non-hematological toxicity of grade ≥3, with the following exceptions:   - Grade 3 fatigue, fever, constipation, nausea, vomiting, or diarrhea lasting <5 days with best supportive care - Grade ≥3 hypertension that can be controlled by medical management - Grade ≥3 ALT or AST that resolves to grade ≤1 or baseline within 7 days, unless criteria for Hy’s law are met^b^ - Isolated grade ≥3 alkaline phosphatase or GGT increase that returns to grade ≤1 or baseline within 7 days - Grade ≥3 lipase or amylase increase not associated with clinical or radiological evidence of pancreatitis - Grade ≥3 electrolyte abnormalities^c^ that resolve spontaneously within 7 days, or that respond to best supportive care - First occurrence of grade ≥3 sARR or CRS | |

Abbreviations: ALP, alkaline phosphatase; ALT, alanine aminotransferase; AST, aspartate aminotransferase; CRS, cytokine release syndrome; DLT, dose-limiting toxicity; GGT, gamma-glutamyl transferase; sARR, systemic administration-related reaction; ULN, upper limit of normal.

^a^Unless unequivocally due to the underlying malignancy or an extraneous cause.

^b^Hy’s law criteria defined as ALT or AST value >3× ULN, total bilirubin >2× ULN, and ALP ≤ 2x ULN, with no alternative etiology. For participants with baseline grade 2 elevation of AST or ALT due to liver metastasis, ALT or AST >3× baseline or AST or ALT >8× ULN, whichever is lower, combined with total bilirubin >2× baseline AND >2× ULN, with no alternative etiology, will be considered as meeting modified Hy’s law.

^c^Any clinically significant chemistry abnormalities grade ≥3 occurring during the DLT period must be reassessed to confirm grade and resolution to grade ≤2.

**Supplementary Table S2.** TEAEs in ≥10% of participants by preferred term: all treated analysis set

| **Participants, *n* (%)** | **All grades** | **Grade ≥3** |
| --- | --- | --- |
| Overall TEAEs | 82 (100.0) | 46 (56.1) |
| TEAEs in ≥10% of participants | | |
| Fatigue | 44 (53.7) | 10 (12.2) |
| Injection site erythema | 41 (50.0) | 0 |
| Decreased appetite | 38 (46.3) | 2 (2.4) |
| Anemia | 30 (36.6) | 14 (17.1) |
| Back pain | 23 (28.0) | 5 (6.1) |
| Weight decreased | 20 (24.4) | 0 |
| Injection site pruritus | 18 (22.0) | 0 |
| Arthralgia | 18 (22.0) | 2 (2.4) |
| Nausea | 17 (20.7) | 1 (1.2) |
| Injection site rash | 14 (17.1) | 0 |
| Pyrexia | 14 (17.1) | 0 |
| Constipation | 14 (17.1) | 0 |
| Dysgeusia | 12 (14.6) | 0 |
| Vomiting | 11 (13.4) | 1 (1.2) |
| Asthenia | 9 (11.0) | 4 (4.9) |
| Abdominal pain | 9 (11.0) | 1 (1.2) |
| AST increased | 9 (11.0) | 2 (2.4) |
| Hypotension | 9 (11.0) | 2 (2.4) |

Abbreviations: AST, Aspartate aminotransferase; TEAE, treatment-emergent adverse event.

A TEAE is defined as any adverse event with onset date and time on or after that of the first dose through 30 days after the last dose of study drug or the day prior to start of subsequent therapy, whichever is earlier.

Participants were counted only once for any given event, regardless of the number of times they experienced the event.

**Supplementary Table S3.** Summarized PK parameters of JNJ-902 after first SC dose (dose 1)

| **Parameters** | **QW SC dosing** | | | | **Q2W SC dosing** | | | |
| --- | --- | --- | --- | --- | --- | --- | --- | --- |
|  | **1.0 mg** | **1.5 mg** | **3.0 mg** | **6.0 mg** | **2.0 mg** | **3.0 mg** | **4.0 mg** | **6.0 mg** |
| *n* | 6 | 12 | 11^a^ | 7 | 4 | 18^b^ | 10 | 6^c^ |
| C_max_, mean (SD), ng/mL | 51.7 (20.6) | 71.0 (28.6) | 129  (74.7) | 249  (130) | 75.6  (13.7) | 116  (56.0) | 131  (47.2) | 329  (109) |
| T_max_, median (range), h | 97.29 (69.50 -  167.67) | 132.00 (70.00 -  192.67) | 72.08 (46.43 -192.92) | 74.00 (67.83 -  164.43) | 169.63 (46.10 -  172.75) | 166.30 (46.08 -  340.10) | 105.09 (45.75 -  186.78) | 165.29 (120.58 -  166.80) |
| AUC_168h_, mean (SD), ng.h/mL, | 6156 (2854) | 9297 (4622) | 17471 (9820) | 31405 (13654) | - | - | - | - |
| AUC_336h_, mean (SD), ng.h/mL | - | - | - | - | 19937 (4469) | 32936 (16708) | 37042 (14102) | 76203 (19107) |

Abbreviations: AUC_Xh_, area under the JNJ-902 serum concentration-time curve from time 0 to X hours; C_max_, maximum JNJ-902 serum concentration; n, number of subjects; QW, once weekly; Q2W, once every two weeks; SC, subcutaneous; SD, standard deviation; T_max_, time to reach maximum JNJ-902 serum concentration.

Mean ± SD are presented if evaluable subjects ≥3. Subjects with a missing JNJ-902 serum concentration around the expected C_max_ were excluded from the descriptive statistics.

0.3 mg SC weekly cohort with an *n* <3 was not shown.

^a^*n* = 9 for AUC_168h._

^b^*n* = 17 for AUC_336h_

^c^*n* = 5 for AUC_336h_

**SupplementaryTable S4.** Summarized PK parameters of JNJ-902 after repeated SC doses (dose 4 and dose 7)

| **Parameters**  mean (SD);  t_max_: median (range) | **Dose 7** | **Dose 4** | | |
| --- | --- | --- | --- | --- |
|  | **1.5 mg QW** | **2.0 mg Q2W** | **3.0 mg Q2W** | **4.0 mg Q2W** |
| *n* | 5^a^ | 3 | 11^b^ | 3 |
| C_min_, mean (SD), ng/mL | 193 (68.9) | 79.2 (8.95) | 165 (74.4) | 78.1 (39.8) |
| C_max_, mean (SD), ng/mL | 242 (112) | 271 (219) | 363 (234) | 202 (92.0) |
| T_max_, median (range), h | 44.50 (23.83 -  47.58) | 71.42 (70.38 -  166.47) | 71.73 (42.75 -  163.58) | 71.28 (30.42 -  75.28) |
| AUC_tau_,  mean (SD),(ng.h/mL | 42849 (15706) | 173281 (232008) | 134623 (60455) | 49394 (22543) |
| RA, mean (SD) | 4.45 (1.52) | 4.09 (3.40) | 3.78 (2.46) | 2.17 (1.52) |
| CL_ss_/F, mean (SD), L/h | 0.0395 (0.0169) | 0.0354 (0.0267) | 0.0265 (0.0124) | 0.0967 (0.0529) |

Abbreviations: AUC_tau_, area under the JNJ-902 serum concentration-time curve from time 0 to the end of dosing interval; C_min_, minimum JNJ-902 serum concentration; C_max_, maximum JNJ-902 serum concentration; CL_ss_/F, total systemic clearance of JNJ-902 following multiple-dose SC administration; QW, once weekly; Q2W, once every two weeks; RA, ratio of accumulation; SC, subcutaneous; SD, standard deviation; T_max_, time to reach maximum JNJ-902 serum concentration.

Mean ± SD are presented if evaluable subjects ≥3. Subjects with a missing JNJ-902 serum concentration around the expected C_max_ were excluded from the descriptive statistics.

PK parameters at dose 7 were not evaluable for 1.0 mg and 6.0 mg SC weekly cohort; 3.0 mg SC weekly cohort with an *n* <3 was not shown.

AUC_tau_ is 168h for QW dosing and 336h for Q2W dosing.

^a^*n* = 4 for AUC_tau_, RA, and CL_ss_/F.

^b^*n* = 5 for AUC_tau_, RA, and CL_ss_/F.
